# Grain Itch

**Published:** 1910-12-31

**Authors:** 


### Medicine.

### GRAIN-ITCH.

Dr. J. F. Schamberg, of Philadelphia, has published a most interesting paper upon an eruption which occurs chiefly in household epidemics, and which is shown to be due to an itch mite derived commonly from the straw employed in making mattresses. It was first noticed in Philadelphia late in the spring of 1901, and it has continued and now also exists in Ohio, Indiana, and Pennsylvania. Microscopical investigations have shown that the eruption is due to a mite. The disease is characterised by a widespread urticaroid eruption accompanied by intense itching, and commonly by mild fever and other general symptoms, including slight leucocytosis, a moderate eosinophilia, and in a few cases albuminuria. The characteristic cutaneous lesion is a wheal surmounted by a vesicle, which rapidly becomes pustular. Up to a certain point the pathological changes of the skin are very similar to those which are characteristic of nettle-rash. The disease is due to contact with cereals or straw infected with a mite of the name of *Pediculoides ventricosus*, though apparently these parasites do not burrow into the skin as does the ordinary common *Sarcoptes hominis*. The most severe and most persistent cases of dermatitis have been traced directly to mattresses, though straw used for packing purposes, wheat, barley, etc., constitute additional sources of infection. The *Pediculoides ventricosus*, from the farmer's point of view, is a beneficial mite, for it has been found to be associated with other insects. It is where grain-destroying insects abound that the mite is present in the largest quantities, its prey being the larvæ of the wheat-straw worm, the joint worm, and the grain moth. An identical or similar affection from contact with barley or wheat has been noticed by naturalists in France, Germany, and Russia, and it may not be unknown in Great Britain, although cases of the kind are not commonly recorded. Once the diagnosis has been arrived at the cure of the condition is easy; the essential point is for the source of the infection to be traced and for the sufferer to avoid re-infection.

